# A new error estimate on uniform norm of Schwarz algorithm for elliptic quasi-variational inequalities with nonlinear source terms

**DOI:** 10.1186/s13660-018-1649-3

**Published:** 2018-03-14

**Authors:** Allaoua Mehri, Samira Saadi

**Affiliations:** 1 Lab. LANOS, Department of Mathematics, University May 8th 1945, Guelma, Algeria; 2 Lab. LANOS, Department of Mathematics, University Badji Mokhtar Annaba, Annaba, Algeria

**Keywords:** 65N30, 65K15, 05C38, 65N15, Schwarz algorithm, Overlapping grids, Quasi-variational inequalities, Stability property, Error estimates

## Abstract

The Schwarz algorithm for a class of elliptic quasi-variational inequalities with nonlinear source terms is studied in this work. The authors prove a new error estimate in uniform norm, making use of a stability property of the discrete solution. The domain is split into two sub-domains with overlapping non-matching grids. This approach combines the geometrical convergence of solutions and the uniform convergence of variational inequalities.

## Introduction

In the present paper, we consider the numerical solution of elliptic quasi-variational inequalities with nonlinear right-hand side. This kind of problem has many applications in impulse control (see [1–4]). The existence, uniqueness, and regularity of the continuous and the discrete solution have been studied and established in the past years (see [3–7]). To estimate a new error of the solution, we apply the Schwarz algorithm, so we split the domain into two overlapping sub-domains such that each sub-domain has its own generated triangulations. In this approach we transform the nonlinear problem into a sequence of linear problems in each sub-domain.

To prove the main result of this paper, we construct two discrete auxiliary sequences of Schwarz, and we estimate the error between continuous and discrete Schwarz iterates. The proof is based on a discrete $L^{\infty }$-stability property with respect to both the boundary condition and the source term for variational inequality, while in [8] the proof is based on a stability property with respect to the boundary condition for variational inequality. Regarding research in this domain, for the linear case we refer the reader to [8–12], and for the nonlinear case we refer to [13–15]. The analysis of geometrical convergence of the Schwarz algorithm has been proven in [8, 16, 17].

This paper consists of two parts. In the first, we formulate the problem of continuous and discrete quasi-variational inequality, we show the monotonicity and stability properties of discrete solution, then we define the Schwarz algorithm for two sub-domains with overlapping non-matching grids. In the second part, we establish two auxiliary Schwarz sequences, and we prove the main result of this work.

## An overlapping Schwarz method for elliptic quasi-variational inequalities with nonlinear source terms

### Formulation of the problem

Let Ω be an open bounded polygon in $R^{2}$ with sufficiently smooth boundary *∂*Ω. We define the bilinear form, for any $u,v\in H^{1}(\Omega)$,

2.1 $$ a(u,v)= \int_{\Omega } \biggl( \sum_{1\leq i,j\leq 2} a_{ij} \frac{\partial u}{\partial x_{i}} \frac{\partial v}{\partial x_{j}} + \sum _{1\leq j \leq 2} a_{j} \frac{\partial u }{\partial x_{j}}v+a_{0} uv \biggr)\,dx, $$

the coefficients $a_{ij}(x)$, $a_{j}(x)$, $a_{0}(x)$ are supposed to be sufficiently smooth and satisfy the following conditions:

2.2 $$\begin{aligned}& \sum_{1\leq i,j\leq 2}a_{ij}(x) \xi_{i}\xi_{j}\geq \alpha \vert \xi \vert ^{2}, \quad \xi \in R^{2},\alpha >0,x\in \overline{\Omega }, \end{aligned}$$

2.3 $$\begin{aligned}& a_{0}(x)\geq \beta >0,\quad \forall x\in \overline{ \Omega }. \end{aligned}$$

We also suppose that the bilinear form is continuous and strongly coercive

2.4 $$ \exists \alpha >0: a(v,v)\geq \alpha \Vert v\Vert _{H^{1}(\Omega)}^{2}. $$

Let the obstacle *Mu* of impulse control be defined by

2.5 $$ Mu(x)=k+\inf u(x+\xi),\quad x\in \Omega, \xi \geq 0, x+\xi \in \Omega, k>0. $$

The operator *M* maps $L^{\infty }(\Omega)$ into itself and possesses the following properties [1]:

2.6 $$\begin{aligned}& Mu\leq M\widetilde{u},\quad \mbox{whenever } u\leq \widetilde{u}, \end{aligned}$$

2.7 $$\begin{aligned}& M(u+c)\leq Mu+c,\quad \mbox{with }c\mbox{ a positive constant} \end{aligned}$$

and a closed convex set

2.8 $$ K_{g}(u)= \bigl\{ v\in H^{1}(\Omega):v=g \mbox{ on } \partial \Omega, v \leq Mu \mbox{ in } \Omega \bigr\} , $$

where *g* is a regular function satisfying

2.9 $$ g\in W^{2,p}(\Omega),\quad 2\leq p< \infty. $$

Let $f(\cdot)$ be the right-hand side supposed nondecreasing and Lipschitz continuous of constant *σ* such that

2.10 $$ \sigma /\beta < 1. $$

We consider the following elliptic quasi-variational inequality (Q.V.I):

2.11 $$ \textstyle\begin{cases} \mbox{find } u\in K_{g}(u)\quad \mbox{solution of } \\ a(u,v-u)\geq (f(u),v-u),\quad \forall v\in K_{g}(u), \end{cases} $$

$(\cdot,\cdot)$ denotes the usual inner product in $L^{2}(\Omega)$.

Thanks to [1], the (QVI) (2.11) has a unique solution; moreover, *u* satisfies the regularity property

$$u\in W^{2,p}(\Omega), \quad 2\leq p< \infty. $$

Let $\tau^{h}$ be a standard regular and quasi-uniform finite element triangulation in Ω, *h* being the mesh size. Let $V_{h}$ denote the standard piecewise linear finite element space. The discrete counterpart of (2.11) consists of

2.12 $$ \textstyle\begin{cases} \mbox{find } u_{h}\in K_{g}^{h}{(u_{h})}\quad \mbox{such that} \\ a(u_{h},v_{h}-u_{h})\geq (f(u_{h}),v_{h}-u_{h}), \quad \forall v_{h}\in K_{g}^{h}({u_{h}}), \end{cases} $$

where

2.13 $$ K_{g}^{h}(u_{h})= \bigl\{ v_{h}\in V_{h}\subset H^{1}(\Omega)/ v_{h}= \pi_{h}g\mbox{ on }\partial \Omega, v_{h} \leq r_{h}Mu_{h}\mbox{ in }\tau^{h} \bigr\} , $$

$r_{h}$ is the usual restriction operator in Ω and $\pi_{h}$ is an interpolation operator on *∂*Ω.

Let $\varphi_{i}$, $i=1,2,\ldots,m(h)$, be basis functions of the space $V_{h}$. We shall assume that the matrix *A* produced by

2.14 $$ A_{ij}=a(\varphi_{i},\varphi_{j}) $$

is *M*-matrix [18].

### Monotonicity and $L^{\infty }$-stability properties

We consider the linear case, for example, $f=f(w)$. Let $(f,g)$, $(\widetilde{f},\widetilde{g})$ be a pair of data of linear functions, and

$$\xi_{h}=\partial_{h}(f, r_{h}M\xi_{h}, \pi_{h}g)\in K_{g}^{h}(\xi_{h}) $$

is the solution of inequality

$$a(\xi_{h},v_{h}-\xi_{h})\geq (f,v_{h}- \xi_{h}),\quad \forall v_{h}\in K _{g}^{h}({ \xi_{h}}), $$

respectively

$$\widetilde{\xi }_{h}=\partial_{h}(\widetilde{f}, r_{h}M \widetilde{\xi }_{h},\pi_{h}\widetilde{g}) \in K_{\widetilde{g}}^{h}( \widetilde{\xi }_{h}) $$

is the solution of inequality

$$a(\widetilde{\xi }_{h},v_{h}-\widetilde{\xi }_{h}) \geq (\widetilde{f},v _{h}-\widetilde{\xi }_{h}),\quad \forall v_{h}\in K_{\widetilde{g}}^{h}( {\widetilde{\xi }_{h}}). $$

Then we give the monotonicity result.

#### Lemma 1

*If* $f\geq \widetilde{f}$, $g\geq \widetilde{g}$, *then* $\partial_{h}(f, r_{h}M\xi_{h}, \pi_{h}g)\geq \partial_{h}(\widetilde{f}, r_{h}M \widetilde{\xi }_{h},\pi_{h}\widetilde{g})$.

#### Proof

Let us reason by recurrence.

For $n=0$: let $\xi_{h}^{0}$ (resp. $\widetilde{\xi }_{h}^{0}$) be the solution of equation

$$\textstyle\begin{cases} a(\xi_{h}^{0},v_{h})=(f,\xi_{h}^{0}),\quad \forall v_{h}\in V_{h}, \\ v_{h}=\pi_{h}g,\quad \mbox{on } \partial \Omega. \end{cases} $$

By the maximum principle, we have

$$\xi_{h}^{0}\geq \widetilde{\xi }_{h}^{0}, $$

and hence by assumption (2.6)

$$r_{h}M\xi_{h}^{0}\geq r_{h}M\widetilde{ \xi }_{h}^{0}, $$

putting

$$\xi_{h}^{1}=\partial_{h} \bigl(f, r_{h}M \xi_{h}^{0}, \pi_{h}g \bigr), $$

(resp.)

$$\widetilde{\xi }_{h}^{1}=\partial_{h} \bigl( \widetilde{f}, r_{h}M \widetilde{\xi }_{h}^{0}, \pi_{h}\widetilde{g} \bigr), $$

applying the monotonicity result for (V.I), we get

$$\xi_{h}^{1}\geq \widetilde{\xi }_{h}^{1}. $$

Now, we define the following sequences:

$$\xi_{h}^{n}=\partial_{h} \bigl(f, r_{h}M \xi_{h}^{n-1}, \pi_{h}g \bigr), $$

(resp.)

$$\widetilde{\xi }_{h}^{n}=\partial_{h} \bigl( \widetilde{f}, r_{h}M \widetilde{\xi }_{h}^{n-1}, \pi_{h}\widetilde{g} \bigr), $$

and we assume that

$$\xi_{h}^{n}\geq \widetilde{\xi }_{h}^{n}. $$

By (2.6), it follows that

$$r_{h}M\xi_{h}^{n}\geq r_{h}M\widetilde{ \xi }_{h}^{n}, $$

therefore, applying again the monotonicity result for (V.I), we obtain

$$\xi_{h}^{n+1}\geq \widetilde{\xi }_{h}^{n+1}. $$

Finally, if $n\longrightarrow \infty $ (see [1]), we get

$$\xi_{h}\geq \widetilde{\xi }_{h}, $$

which concludes the proof. □

The proposition below establishes an $L^{\infty }$-stability property of the solution with respect to the data.

#### Proposition 1

*Under conditions of Lemma *1, *we have*

$$\bigl\Vert \partial_{h}(f, r_{h}M\xi_{h}, \pi_{h}g)- \partial_{h}(\widetilde{f},r_{h}M \widetilde{\xi }_{h},\pi_{h}\widetilde{g}) \bigr\Vert _{L ^{\infty }(\Omega)}\leq \max \biggl\{ \frac{1}{\beta } \Vert f-\widetilde{f} \Vert _{L^{\infty }(\Omega)}, \Vert g-\widetilde{g}\Vert _{L^{ \infty }(\partial \Omega)} \biggr\} . $$

#### Proof

Firstly, set

$$\Phi =\max \biggl\{ \frac{1}{\beta }\Vert f-\widetilde{f}\Vert _{L^{\infty }( \Omega)}, \Vert g-\widetilde{g}\Vert _{L^{\infty }(\partial \Omega)} \biggr\} , $$

we have

$$a(\widetilde{\xi }_{h}, v_{h}-\widetilde{\xi }_{h})\geq ( \widetilde{f}, v_{h}-\widetilde{\xi }_{h}) $$

and

$$a(\Phi, v_{h}-\widetilde{\xi }_{h})=\Phi (a_{0}, v_{h}- \widetilde{\xi }_{h}). $$

By summation, we get

$$a(\widetilde{\xi }_{h}+\Phi, v_{h}-\widetilde{\xi }_{h})\geq ( \widetilde{f}+a_{0}\Phi, v_{h}- \widetilde{\xi }_{h}) $$

and

$$a \bigl(\widetilde{\xi }_{h}+\Phi, (v_{h}+\Phi)-( \widetilde{\xi }_{h}+ \Phi) \bigr)\geq \bigl(\widetilde{f}+a_{0} \Phi, (v_{h}+\Phi)-( \widetilde{\xi }_{h}+\Phi) \bigr). $$

If we put

$$\overline{\xi }_{h}=\widetilde{\xi }_{h}+\Phi,\qquad \overline{v}_{h}=v _{h}+\Phi, $$

then

$$a(\overline{\xi }_{h},\overline{v}_{h}-\overline{\xi }_{h})\geq ( \widetilde{f}+a_{0}\Phi, \overline{v}_{h}- \overline{\xi }_{h}), $$

therefore

$$\overline{\xi }_{h}=\partial_{h} \bigl(\widetilde{f}+a_{0} \Phi, r_{h}M\overline{ \xi }_{h},\pi_{h}( \widetilde{g}+\Phi) \bigr), $$

where

$$\overline{\xi }_{h}\leq r_{h}M\overline{\xi }_{h}=r_{h}M( \widetilde{\xi }_{h}+\Phi). $$

By (2.7), it follows that

$$\widetilde{\xi }_{h}\leq r_{h}M\widetilde{\xi }_{h}, $$

so

$$\partial_{h} \bigl(\widetilde{f}+a_{0}\Phi, r_{h}M \overline{\xi }_{h},\pi _{h}(\widetilde{g}+\Phi) \bigr) = \partial_{h}(\widetilde{f}, r_{h}M \widetilde{\xi }_{h},\pi_{h}\widetilde{g})+\Phi. $$

Secondly, we have

$$\begin{aligned} f \leq & \widetilde{f}+ \Vert f-\widetilde{f}\Vert _{L^{\infty }(\Omega)} \\ \leq & \widetilde{f}+ \frac{a_{0}}{\beta }\Vert f-\widetilde{f}\Vert _{L^{ \infty }(\Omega)} \\ \leq & \widetilde{f}+ a_{0}\Phi \end{aligned}$$

and

$$\begin{aligned} g \leq & \widetilde{g}+\Vert g-\widetilde{g}\Vert _{L^{\infty }(\partial \Omega)} \\ \leq & \widetilde{g}+\Phi. \end{aligned}$$

Using Lemma 1, we get

$$\begin{aligned} \partial_{h}(f, r_{h}M\xi_{h}, \pi_{h}g) \leq & \partial_{h} \bigl( \widetilde{f}+a_{0}\Phi, r_{h}M(\widetilde{\xi }_{h}+\Phi),\pi_{h}( \widetilde{g}+\Phi) \bigr) \\ =& \partial_{h}(\widetilde{f}, r_{h}M\widetilde{\xi }_{h},\pi_{h} \widetilde{g})+\Phi, \end{aligned}$$

then

$$\xi_{h}\leq \widetilde{\xi }_{h}+\Phi. $$

Similarly, interchanging the roles of the couples $(f,g) $ and $(\widetilde{f},\widetilde{g})$, we obtain

$$\widetilde{\xi }_{h}\leq \xi_{h}+\Phi, $$

which completes the proof. □

The following result is due to [6].

#### Theorem 1

*There exists a constant* *c* *independent of* *h* *such that*

$$\bigl\Vert \partial_{h}(f, r_{h}M\xi_{h}, \pi_{h}g)-\partial (f, M\xi, g) \bigr\Vert _{L^{\infty }(\Omega)} \leq ch^{2}\vert \log h\vert ^{2}. $$

### The continuous Schwarz algorithm

We consider the problem: find $u\in K_{0}(u)$ such that

2.15 $$ a(u,v-u)\geq \bigl(f(u),v-u \bigr),\quad \forall v\in K_{0}(u), $$

where $K_{0}(u)$ is defined in (2.8) with $g=0$.

We split Ω into two overlapping polygonal sub-domains $\Omega_{1}$ and $\Omega_{2}$ such that

$$\Omega_{1}\cap \Omega_{2}\neq \emptyset,\qquad \Omega = \Omega_{1}\cup \Omega_{2}, $$

and *u* satisfies the local regularity condition

$$u_{\vert \Omega_{i}}\in W^{2,p}(\Omega_{i}),\quad 2\leq p< \infty. $$

We set $\Gamma_{i}=\partial \Omega_{i}\cap \Omega_{j}$, where $\partial \Omega_{i}$ denotes the boundary of $\Omega_{i}$. The intersection of $\Gamma_{1}$ and $\Gamma_{2}$ is assumed to be empty. We will always assume to simplify that $\Gamma_{1}$, $\Gamma_{2}$ are smooth.

For $w\in C^{0}(\overline{\Gamma }_{i})$, we define

$$V_{i}^{(w)}= \bigl\{ v\in H^{1}( \Omega_{i}) / v=0 \mbox{ on } \partial \Omega \cap \partial \Omega_{i} , v=w \mbox{ on } \Gamma _{i} \bigr\} , \quad i=1,2. $$

We associate with problem (2.15) the couple $(u_{1},u_{2}) \in V_{1}^{(u_{2})}\times V_{2}^{(u_{1})}$ such that

2.16 $$\begin{aligned}& \textstyle\begin{cases} a_{1}(u_{1},v-u_{1})\geq (f(u_{1}),v-u_{1}),\quad \forall v\in V_{1}^{(u _{2})}, \\ u_{1}\leq Mu_{1} ,\qquad v\leq Mu_{1} \quad \mbox{in } \Omega_{1}, \end{cases}\displaystyle \end{aligned}$$

2.17 $$\begin{aligned}& \textstyle\begin{cases} a_{2}(u_{2},v-u_{2})\geq (f(u_{2}),v-u_{2}),\quad \forall v\in V_{2}^{(u _{1})}, \\ u_{2}\leq Mu_{2} ,\qquad v\leq Mu_{2}\quad \mbox{in } \Omega_{2}, \end{cases}\displaystyle \end{aligned}$$

where

$$\begin{aligned}& a_{i}(u,v)= \int_{\Omega_{i}} \biggl( \sum_{1\leq l,j\leq 2} a_{lj} \frac{ \partial u}{\partial x_{l}} \frac{\partial v}{\partial x_{j}} + \sum _{1\leq j \leq 2} a_{j} \frac{\partial u }{\partial x_{j}}v+a_{0} uv \biggr)\,dx,\quad i=1,2, \\& u_{i} =u_{\vert \Omega_{i} },\quad i=1,2. \end{aligned}$$

Let $u^{0}\in C^{0}(\overline{\Omega })$ be the initial value such that

2.18 $$ a \bigl(u^{0},v \bigr)= \bigl(f \bigl(u^{0} \bigr),v \bigr),\quad \forall v\in H_{0}^{1}(\Omega). $$

We define the Schwarz sequence $(u_{1}^{n+1})$ on $\Omega_{1}$ such that $u_{1}^{n+1}\in V_{1}^{(u_{2}^{n})}$ solves

2.19 $$ \textstyle\begin{cases} a_{1}(u_{1}^{n+1},v-u_{1}^{n+1})\geq (f(u_{1}^{n}),v-u_{1}^{n+1}),\quad \forall v\in V_{1}^{(u_{2}^{n})}, \\ u_{1}^{n+1}\leq Mu_{1}^{n} ,\qquad v\leq Mu_{1}^{n} \quad \mbox{in } \Omega_{1}, \end{cases} $$

and respectively $(u_{2}^{n+1})$ on $\Omega_{2}$ such that $u_{2}^{n+1} \in V_{2}^{(u_{1}^{n})}$ solves

2.20 $$ \textstyle\begin{cases} a_{2}(u_{2}^{n+1},v-u_{2}^{n+1})\geq (f(u_{2}^{n}),v-u_{2}^{n+1}),\quad \forall v\in V_{2}^{(u_{1}^{n})}, \\ u_{2}^{n+1}\leq Mu_{2}^{n} ,\qquad v\leq Mu_{2}^{n} \quad \mbox{in } \Omega_{2}, \end{cases} $$

where

$$\begin{aligned}& u_{1}^{0} = u^{0}\quad \mbox{in } \Omega_{1},\qquad u_{2}^{0}=u^{0} \quad \mbox{in }\Omega_{2}, \\& u_{1}^{n+1} = 0\quad \mbox{in }\overline{\Omega }\setminus \overline{ \Omega }_{1},\qquad u_{2}^{n+1}=0\quad \mbox{in }\overline{\Omega } \setminus \overline{\Omega }_{2}. \end{aligned}$$

We give a geometrical convergence theorem (see [8]).

#### Theorem 2

*The sequences* $(u_{1}^{n+1}, u_{2}^{n+1})$, $n\geq 0$ *converge geometrically to the solution* $(u_{1}, u_{2})$ *of system* (2.16)*–*(2.17). *More precisely*, *there exist two constants* $0< k_{1},k_{2}<1 $ *such that*

$$\begin{aligned}& \bigl\Vert u_{1}-u_{1}^{n+1} \bigr\Vert _{L^{\infty }(\Omega_{1})} \leq k_{1}^{n}k_{2}^{n} \bigl\Vert u^{0}-u \bigr\Vert _{L^{\infty }( \Gamma_{1})}, \\& \bigl\Vert u_{2}-u_{2}^{n+1} \bigr\Vert _{L^{\infty }(\Omega_{2})} \leq k_{1}^{n}k_{2}^{n} \bigl\Vert u^{0}-u \bigr\Vert _{L^{\infty }( \Gamma_{2})}. \end{aligned}$$

### The discretization

Let $\tau^{h_{i}}$ be a standard regular and quasi-uniform finite element triangulation in $\Omega_{i}$; $i=1,2$, $h_{i}$ being the mesh size. We assume that $\tau^{h_{1}}$ and $\tau^{h_{2}}$ are mutually independent on $\Omega_{1}\cap \Omega_{2}$, in the sense that a triangle belonging to $\tau^{h_{i}}$ does not necessarily belong to $\tau^{h _{j}}$, $i\neq j$. Let $V_{h_{i}}=V_{h_{i}}(\Omega_{i})$ be the space of continuous piecewise linear functions on $\tau^{h_{i}}$ which vanish on $\partial \Omega \cap \partial \Omega_{i}$. For given $w\in C^{0}(\overline{ \Gamma }_{i})$, we set

$$V_{h_{i}}^{(w)}= \bigl\{ v_{h_{i}}\in V_{h_{i}} / v_{h _{i}}=\pi_{h_{i}}(w) \mbox{ on } \Gamma_{i} \bigr\} ,\quad i=1,2, $$

where $\pi_{h_{i}}$ denotes a suitable interpolation operator on $\Gamma_{i}$. We give the discrete counterpart of the Schwarz algorithm defined in (2.19) and (2.20) as follows.

Let $u_{h_{i}}^{0}=r_{h_{i}}u^{0}$ be given, we define the discrete Schwarz sequence $(u_{1h_{1}}^{n+1})$ on $\Omega_{1}$ such that $u_{1h_{1}}^{n+1}\in V_{h_{1}}^{(u_{2h_{2}}^{n})}$ solves

2.21 $$ \textstyle\begin{cases} a_{1}(u_{1h_{1}}^{n+1},v_{h_{1}}-u_{1h_{1}}^{n+1})\geq (f(u_{1h_{1}} ^{n}),v_{h_{1}}-u_{1h_{1}}^{n+1}),\quad \forall v_{h_{1}}\in V_{h_{1}}^{(u _{2h_{2}}^{n})}, \\ u_{1h_{1}}^{n+1}\leq r_{h_{1}}Mu_{1h_{1}}^{n} v_{h_{1}}\leq r_{h_{1}}Mu_{1h_{1}}^{n} \quad \mbox{in } \tau^{h_{1}}, \end{cases} $$

and on $\Omega_{2}$ the sequence $u_{2h_{2}}^{n+1}\in V_{h_{2}}^{(u _{1h_{1}}^{n})}$ solves

2.22 $$ \textstyle\begin{cases} a_{2}(u_{2h_{2}}^{n+1},v_{h_{2}}-u_{2h_{2}}^{n+1})\geq (f(u_{2h_{2}} ^{n}),v_{h_{2}}-u_{2h_{2}}^{n+1}),\quad \forall v_{h_{2}}\in V_{h_{2}}^{(u _{1h_{1}}^{n})}, \\ u_{2h_{2}}^{n+1}\leq r_{h_{2}}Mu_{2h_{2}}^{n} v_{h_{2}}\leq r_{h_{2}}Mu_{2h_{2}}^{n} \quad \mbox{in } \tau^{h_{2}}, \end{cases} $$

with

$$u_{1h_{1}}^{0}=u_{h_{1}}^{0} \quad \mbox{in } \Omega_{1}, \qquad u_{2h _{2}}^{0}=u_{h_{2}}^{0} \quad \mbox{in }\Omega_{2}. $$

We will also assume that the respective matrices produced by problems (2.21) and (2.22) are *M*-matrices [18].

## $L^{\infty }$-error analysis

The aim of this section is to show the main result of this paper. To that end, we start by introducing two discrete auxiliary sequences and prove a fundamental lemma.

### Two discrete auxiliary sequences

For $w_{ih_{i}}^{0}=u_{h_{i}}^{0}$, we define the sequence $w_{1h_{1}} ^{n+1}\in V_{h_{1}}^{(u_{2}^{n})}$, discrete solution of V.I

3.1 $$ \textstyle\begin{cases} a_{1}(w_{1h_{1}}^{n+1},v_{h_{1}}-w_{1h_{1}}^{n+1})\geq (f(u_{1}^{n}),v _{h_{1}}-w_{1h_{1}}^{n+1}),\quad \forall v_{h_{1}}\in V_{h_{1}}^{(u_{2}^{n})}, \\ w_{1h_{1}}^{n+1}\leq r_{h_{1}}Mw_{1h_{1}}^{n} v_{h_{1}}\leq r_{h_{1}}Mw_{1h_{1}}^{n} \quad \mbox{in } \tau^{h_{1}}, \end{cases} $$

respectively the sequence $w_{2h_{2}}^{n+1}\in V_{h_{2}}^{(u_{1}^{n})}$ satisfies

3.2 $$ \textstyle\begin{cases} a_{2}(w_{2h_{2}}^{n+1},v_{h_{2}}-w_{2h_{2}}^{n+1})\geq (f(u_{2}^{n}),v _{h_{2}}-w_{2h_{2}}^{n+1}),\quad \forall v_{h_{2}}\in V_{h_{2}}^{(u_{1}^{n})}, \\ w_{2h_{2}}^{n+1}\leq r_{h_{2}}Mw_{2h_{2}}^{n} v_{h_{2}}\leq r_{h_{2}}Mw_{2h_{2}}^{n} \quad \mbox{in } \tau^{h_{2}}. \end{cases} $$

To simplify the notation, we take

$$\begin{aligned}& \vert \cdot \vert _{1}=\Vert \cdot \Vert _{L^{\infty }(\Gamma_{1})},\qquad \vert \cdot \vert _{2}=\Vert \cdot \Vert _{L^{\infty }(\Gamma_{2})}, \\& \Vert \cdot \Vert _{1}=\Vert \cdot \Vert _{L^{\infty }(\Omega_{1})},\qquad \Vert \cdot \Vert _{2}= \Vert \cdot \Vert _{L^{\infty }(\Omega_{2})}, \\& h_{1}=h_{2}=h,\qquad r_{h_{1}}=r_{h_{2}}=r_{h}, \qquad \pi_{h _{1}}=\pi_{h_{2}}=\pi_{h}. \end{aligned}$$

It is clear that $w_{ih_{i}}^{n}$, $i=1,2$, is the finite element approximations of $u_{i}^{n}$ defined in (2.19), (2.20), respectively, where $f(\cdot)$ is Lipschitz continuous and $\Vert f(u_{i}^{n}\Vert _{i}\leq c$ (independent of *n*).

The following lemma will play a crucial role in proving the main result of this paper.

#### Lemma 2

*Let* $(u_{i}^{n+1})$, $(u_{ih}^{n+1})$, $i= 1,2$, *be the respective sequences defined in* (2.19), (2.20), (2.21), *and* (2.22). *Then there exists a constant* *c* *independent of* *h* *and* *n* *such that*

$$\bigl\Vert u_{i}^{n+1}-u_{ih}^{n+1} \bigr\Vert _{i} \leq c(n+1)h^{2}\vert \log h\vert ^{2},\quad i=1,2. $$

#### Proof

Let $\theta =\sigma /\beta $, under assumption (2.10), we have

$$\theta < 1. $$

Let us prove by inductionfor $n=0$:

$$\bigl\Vert u_{1}^{1}-u_{1h}^{1} \bigr\Vert _{1}\leq \bigl\Vert u_{1}^{1}-w_{1h}^{1} \bigr\Vert _{1}+ \bigl\Vert w_{1h}^{1}-u_{1h}^{1} \bigr\Vert _{1}. $$

Applying Theorem 1 and Proposition 1, putting $f=f(u_{1}^{0})$, $\widetilde{f}=f(u_{1h}^{0})$, we obtain

$$\begin{aligned} \bigl\Vert u_{1}^{1}-u_{1h}^{1} \bigr\Vert _{1} \leq & ch^{2}\vert \log h\vert ^{2}+ \max \biggl\{ \frac{1}{ \beta } \bigl\Vert f \bigl(u_{1}^{0} \bigr)-f \bigl(u_{1h}^{0} \bigr) \bigr\Vert _{1}, \bigl\vert u_{2}^{0}-u_{2h}^{0} \bigr\vert _{1} \biggr\} \\ \leq & ch^{2}\vert \log h\vert ^{2}+ \max \bigl\{ \theta \bigl\Vert u_{1}^{0}-u_{1h}^{0} \bigr\Vert _{1}, \bigl\vert u_{2}^{0}-u_{2h}^{0} \bigr\vert _{1} \bigr\} . \end{aligned}$$

If

$$\max \bigl\{ \theta \bigl\Vert u_{1}^{0}-u_{1h}^{0} \bigr\Vert _{1}, \bigl\vert u_{2}^{0}-u_{2h}^{0} \bigr\vert _{1} \bigr\} =\theta \bigl\Vert u_{1}^{0}-u_{1h}^{0} \bigr\Vert _{1}, $$

then

$$\begin{aligned} \bigl\Vert u_{1}^{1}-u_{1h}^{1} \bigr\Vert _{1} \leq & ch^{2}\vert \log h\vert ^{2}+ \theta \bigl\Vert u_{1}^{0}-u_{1h}^{0} \bigr\Vert _{1} \\ \leq & ch^{2}\vert \log h\vert ^{2}+ \bigl\Vert u_{1}^{0}-u_{1h}^{0} \bigr\Vert _{1}. \end{aligned}$$

Making use of an error estimate for elliptic variational equations [19], we obtain

$$\bigl\Vert u_{1}^{1}-u_{1h}^{1} \bigr\Vert _{1} \leq ch^{2}\vert \log h\vert ^{2}+ch^{2}\vert \log h\vert \leq ch^{2}\vert \log h\vert ^{2}, $$

and if

$$\max \bigl\{ \theta \bigl\Vert u_{1}^{0}-u_{1h}^{0} \bigr\Vert _{1}, \bigl\vert u_{2}^{0}-u_{2h}^{0} \bigr\vert _{1} \bigr\} = \bigl\vert u_{2}^{0}-u_{2h}^{0} \bigr\vert _{1}, $$

then

$$\begin{aligned} \bigl\Vert u_{1}^{1}-u_{1h}^{1} \bigr\Vert _{1} \leq & ch^{2}\vert \log h\vert ^{2}+ \bigl\vert u_{2}^{0}-u_{2h}^{0} \bigr\vert _{1} \\ \leq & ch^{2}\vert \log h\vert ^{2}+ \bigl\Vert u_{2}^{0}-u_{2h}^{0} \bigr\Vert _{2}. \end{aligned}$$

Making use again of an error estimate for elliptic variational equations [19], we obtain

$$\begin{aligned} \bigl\Vert u_{1}^{1}-u_{1h}^{1} \bigr\Vert _{1} \leq & ch^{2}\vert \log h\vert ^{2}+ ch^{2}\vert \log h\vert \\ \leq & ch^{2}\vert \log h\vert ^{2}. \end{aligned}$$

Similarly, we have in domain $\Omega_{2}$

$$\begin{aligned} \bigl\Vert u_{2}^{1}-u_{2h}^{1} \bigr\Vert _{2} \leq & \bigl\Vert u_{2}^{1}-w_{2h}^{1} \bigr\Vert _{2}+ \bigl\Vert w_{2h}^{1}-u_{2h}^{1} \bigr\Vert _{2} \\ \leq & ch^{2}\vert \log h\vert ^{2}+ \max \biggl\{ \frac{1}{\beta } \bigl\Vert f \bigl(u_{2}^{0} \bigr)-f \bigl(u_{2h}^{0} \bigr) \bigr\Vert _{2}, \bigl\vert u_{1}^{0}-u_{1h}^{0} \bigr\vert _{2} \biggr\} \\ \leq & ch^{2}\vert \log h\vert ^{2}+ \max \bigl\{ \theta \bigl\Vert u_{2}^{0}-u_{2h}^{0} \bigr\Vert _{2}, \bigl\vert u_{1}^{0}-u_{1h}^{0} \bigr\vert _{2} \bigr\} . \end{aligned}$$

If

$$\max \bigl\{ \theta \bigl\Vert u_{2}^{0}-u_{2h}^{0} \bigr\Vert _{2}, \bigl\vert u_{1}^{0}-u_{1h}^{0} \bigr\vert _{2} \bigr\} = \theta \bigl\Vert u_{2}^{0}-u_{2h}^{0} \bigr\Vert _{2}, $$

therefore

$$\begin{aligned} \bigl\Vert u_{2}^{1}-u_{2h}^{1} \bigr\Vert _{2} \leq & ch^{2}\vert \log h\vert ^{2}+ \theta \bigl\Vert u_{2}^{0}-u_{2h}^{0} \bigr\Vert _{2} \\ \leq & ch^{2}\vert \log h\vert ^{2}+ \bigl\Vert u_{2}^{0}-u_{2h}^{0} \bigr\Vert _{2} \\ \leq & ch^{2}\vert \log h\vert ^{2}+ ch^{2} \vert \log h\vert \\ \leq & ch^{2}\vert \log h\vert ^{2}, \end{aligned}$$

and if

$$\max \bigl\{ \theta \bigl\Vert u_{2}^{0}-u_{2h}^{0} \bigr\Vert _{2}, \bigl\vert u_{1}^{0}-u_{1h}^{0} \bigr\vert _{2} \bigr\} = \bigl\vert u_{1}^{0}-u_{1h}^{0} \bigr\vert _{2}, $$

then

$$\begin{aligned} \bigl\Vert u_{2}^{1}-u_{2h}^{1} \bigr\Vert _{2} \leq & ch^{2}\vert \log h\vert ^{2}+ \bigl\vert u_{1}^{0}-u_{1h}^{0} \bigr\vert _{2} \\ \leq & ch^{2}\vert \log h\vert ^{2}+ \bigl\Vert u_{1}^{0}-u_{1h}^{0} \bigr\Vert _{1} \\ \leq & ch^{2}\vert \log h\vert ^{2}+ ch^{2} \vert \log h\vert \\ \leq & ch^{2}\vert \log h\vert ^{2}. \end{aligned}$$

Let us now assume that

$$\bigl\Vert u_{1}^{n}-u_{1h}^{n} \bigr\Vert _{1} \leq cnh^{2}\vert \log h\vert ^{2} $$

and

$$\bigl\Vert u_{2}^{n}-u_{2h}^{n} \bigr\Vert _{2} \leq cnh^{2}\vert \log h\vert ^{2}. $$

Consequently,

$$\begin{aligned} \bigl\Vert u_{1}^{n+1}-u_{1h}^{n+1} \bigr\Vert _{1} \leq & \bigl\Vert u_{1}^{n+1}-w_{1h}^{n+1} \bigr\Vert _{1}+ \bigl\Vert w_{1h}^{n+1}-u_{1h}^{n+1} \bigr\Vert _{1} \\ \leq & ch^{2}\vert \log h\vert ^{2}+ \max \biggl\{ \frac{1}{\beta } \bigl\Vert f \bigl(u_{1}^{n} \bigr)-f \bigl(u_{1h}^{n} \bigr) \bigr\Vert _{1}, \bigl\vert u_{2}^{n}-u_{2h}^{n} \bigr\vert _{1} \biggr\} \\ \leq & ch^{2}\vert \log h\vert ^{2}+ \max \bigl\{ \theta \bigl\Vert u_{1}^{n}-u_{1h}^{n} \bigr\Vert _{1}, \bigl\vert u_{2}^{n}-u_{2h}^{n} \bigr\vert _{1} \bigr\} . \end{aligned}$$

If

$$\max \bigl\{ \theta \bigl\Vert u_{1}^{n}-u_{1h}^{n} \bigr\Vert _{1}, \bigl\vert u_{2}^{n}-u_{2h}^{n} \bigr\vert _{1} \bigr\} =\theta \bigl\Vert u_{1}^{n}-u_{1h}^{n} \bigr\Vert _{1}, $$

then

$$\begin{aligned} \bigl\Vert u_{1}^{n+1}-u_{1h}^{n+1} \bigr\Vert _{1} \leq & ch^{2}\vert \log h\vert ^{2}+ \theta \bigl\Vert u_{1}^{n}-u_{1h}^{n} \bigr\Vert _{1} \\ \leq & ch^{2}\vert \log h\vert ^{2}+ \bigl\Vert u_{1}^{n}-u_{1h}^{n} \bigr\Vert _{1} \\ \leq & ch^{2}\vert \log h\vert ^{2}+ cnh^{2} \vert \log h\vert ^{2} \\ \leq & c(n+1)h^{2}\vert \log h\vert ^{2}, \end{aligned}$$

and if

$$\max \bigl\{ \theta \bigl\Vert u_{1}^{n}-u_{1h}^{n} \bigr\Vert _{1}, \bigl\vert u_{2}^{n}-u_{2h}^{n} \bigr\vert _{1} \bigr\} = \bigl\vert u_{2}^{n}-u_{2h}^{n} \bigr\vert _{1}, $$

therefore

$$\begin{aligned} \bigl\Vert u_{1}^{n+1}-u_{1h}^{n+1} \bigr\Vert _{1} \leq & ch^{2}\vert \log h\vert ^{2}+ \bigl\vert u_{2}^{n}-u_{2h}^{n} \bigr\vert _{1} \\ \leq & ch^{2}\vert \log h\vert ^{2}+ \bigl\Vert u_{2}^{n}-u_{2h}^{n} \bigr\Vert _{2} \\ \leq & ch^{2}\vert \log h\vert ^{2}+ cnh^{2} \vert \log h\vert ^{2} \\ \leq & c(n+1)h^{2}\vert \log h\vert ^{2}. \end{aligned}$$

Similarly, we prove the estimate in domain $\Omega_{2}$. □

### $L^{\infty }$-error estimate

#### Theorem 3

(Main result)

*Let* $(u_{i}^{n+1})$, $(u_{ih}^{n+1})$, $i=1,2$, *be the respective solutions of* (2.19), (2.20), (2.21), *and* (2.22). *Then*, *for* *n* *large enough*, *there exists a constant* *c* *independent of* *h* *and* *n* *such that*

$$\begin{aligned}& \bigl\Vert u_{i}-u_{ih}^{n+1} \bigr\Vert _{i} \leq ch^{2}\vert \log h\vert ^{3} , \\& \bigl\Vert u_{i}-u_{ih}^{n+1} \bigr\Vert _{W^{1,\infty }(\Omega_{i})} \leq ch\vert \log h\vert ^{3} . \end{aligned}$$

#### Proof

Let us give the proof for $i= 1$. The case $i = 2$ is similar.

Indeed, let $k=\max (k_{1},k_{2})$. It follows from Theorem 2 and Lemma 2 that

$$\begin{aligned} \bigl\Vert u_{1}-u_{1h}^{n+1} \bigr\Vert _{1} \leq & \bigl\Vert u_{1}-u_{1}^{n+1} \bigr\Vert _{1}+ \bigl\Vert u_{1}^{n+1}-u_{1h}^{n+1} \bigr\Vert _{1} \\ \leq & k_{1}^{n}k_{2}^{n} \bigl\vert u-u^{0} \bigr\vert _{1}+c(n+1)h^{2}\vert \log h \vert ^{2} \\ \leq & \bigl\vert u-u^{0} \bigr\vert _{1}+c(n+1)h^{2} \vert \log h\vert ^{2} \\ \leq & ch^{2}\vert \log h\vert ^{2}+ c(n+1)h^{2}\vert \log h\vert ^{2}. \end{aligned}$$

We choose *n* such that

$$k^{n}\geq h, $$

then

$$\begin{aligned} \bigl\Vert u_{1}-u_{1h}^{n+1} \bigr\Vert _{1} \leq & ch^{2}\vert \log h\vert ^{3}, \end{aligned}$$

and by inverse inequality, we get

$$\bigl\Vert u_{1}-u_{1h}^{n+1}\bigr\Vert _{W^{1,\infty }(\Omega_{1})} \leq ch\vert \log h\vert ^{3}, $$

which is the desired error estimate. □

## Conclusion

In this work, we have established a new approach of an overlapping Schwarz algorithm on non-matching grids for a class of elliptic quasi-variational inequalities with nonlinear source terms. We have obtained a new error estimate in uniform norm which is optimal for these problems. The error estimate obtained contains a logarithmic factor with an extra power of $\vert \log h\vert $ than expected. We will see that this result plays an important role in the study of an error estimate for evolutionary problems with nonlinear source terms.
